# A parametric study of the effect of 3D plaque shape on local hemodynamics and implications for plaque instability

**DOI:** 10.1007/s10237-024-01834-6

**Published:** 2024-03-26

**Authors:** Shaolie S. Hossain, Michael J. Johnson, Thomas J. R. Hughes

**Affiliations:** 1 Molecular Cardiology Research Laboratories, The Texas Heart Institute, 6770 Bertner Avenue, Houston, TX 77030 USA; 2 Oden Institute for Computational Engineering and Sciences, The University of Texas at Austin, 201 E. 24th St, Austin, TX 78712 USA

**Keywords:** Vulnerable plaque, Patient-specific modeling, Wall shear stress gradient, Inflammation, Atherosclerosis, Isogeometric analysis

## Abstract

The vast majority of heart attacks occur when vulnerable plaques rupture, releasing their lipid content into the blood stream leading to thrombus formation and blockage of a coronary artery. Detection of these unstable plaques before they rupture remains a challenge. Hemodynamic features including wall shear stress (WSS) and wall shear stress gradient (WSSG) near the vulnerable plaque and local inflammation are known to affect plaque instability. In this work, a computational workflow has been developed to enable a comprehensive parametric study detailing the effects of 3D plaque shape on local hemodynamics and their implications for plaque instability. Parameterized geometric 3D plaque models are created within a patient-specific coronary artery tree using a NURBS (non-uniform rational B-splines)-based vascular modeling pipeline. Realistic blood flow features are simulated by using a Navier–Stokes solver within an isogeometric finite-element analysis framework. Near wall hemodynamic quantities such as WSS and WSSG are quantified, and vascular distribution of an inflammatory marker (VCAM-1) is estimated. Results show that proximally skewed eccentric plaques have the most vulnerable combination of high WSS and high positive spatial WSSG, and the presence of multiple lesions increases risk of rupture. The computational tool developed in this work, in conjunction with clinical data, -could help identify surrogate markers of plaque instability, potentially leading to a noninvasive clinical procedure for the detection of vulnerable plaques before rupture.

## Introduction

### Vulnerable plaques and heart attacks

**Fig. 1:**
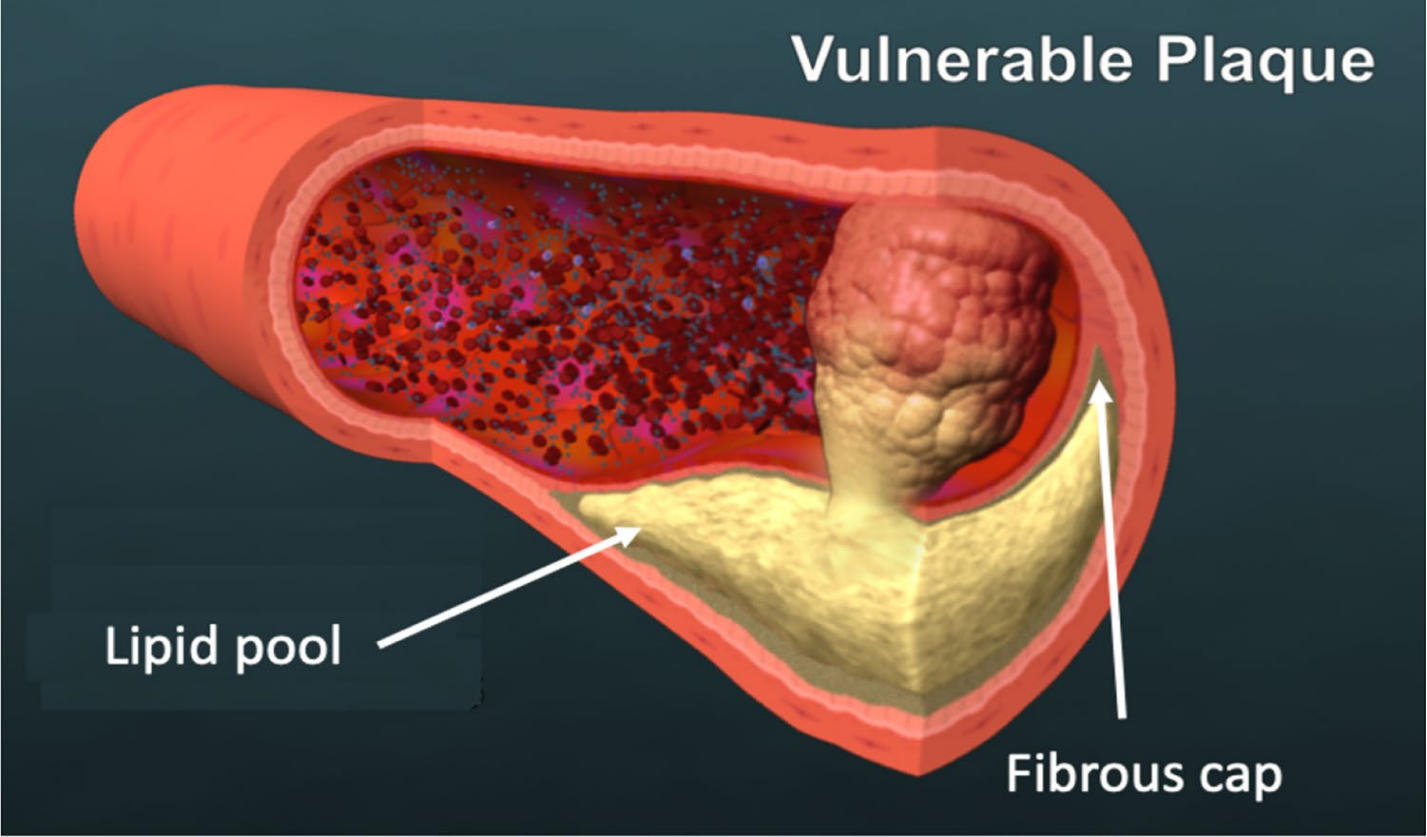
A schematic of a vulnerable plaque. Vulnerable plaques are typically characterized by a large lipid core encased by a thin fibrous cap. As the plaque ruptures and exposes its thrombogenic contents into the blood stream, blood clots are formed. This can occlude the blood vessel leading to myocardial infarction or heart attack

Roughly 70% heart attacks occur when unstable atherosclerotic plaques, so called vulnerable plaques, rupture and release their thrombogenic content into the bloodstream forming a thrombus (Fig. 1). This restricts blood flow to the heart muscle, inducing myocardial infarction (MI) or heart attack. Vulnerable plaques are typically characterized by a large lipid core with abundant inflammatory cells, encased by a thin fibrous cap that is formed just under the endothelium at the blood interface. Because vulnerable plaques generally do not create significant narrowing of the artery (Glaser et al. 2005), they often go undetected by conventional imaging that provide direct visualization and measurement of vessel caliber such as coronary angiography. While optical coherence tomography (OCT) and intravascular ultrasound (IVUS) imaging are able to show plaque composition and are often able to distinguish unstable plaques from stable plaques, these invasive imaging modalities are not typically used in clinic. As a result, detection of vulnerable plaques remains a huge unmet clinical need. The ability to noninvasively identify unstable plaques before rupture could improve risk stratification and enable preventive intervention.

### Factors influencing plaque instability

Plaques rupture when mechanical stress exceeds the material strength of the overlying fibrous cap (Costopoulos et al. 2017). Factors that influence plaque instability can thus be divided into two categories: (1) factors that can increase the intraplaque stress (e.g., a thin fibrous cap, a large lipid pool, and less stenotic lesions) (Alegre-Martínez et al. 2019) and (2) factors that can indirectly weaken the fibrous cap (e.g., inflammation, decreased smooth muscle cells, collagen degradation) (Lee and Libby 1997). Plaque destabilization due to weakening of the fibrous cap originates from the dysfunction of the endothelium overlaying a vascular lesion. This dysfunction, commonly referred to as endothelial activation, signifies a proinflammatory shift from a quiescent phenotype. Local hemodynamics, which is known to regulate endothelial behavior by influencing endothelial cell (EC) shape, intracellular signalling, and gene expression, has the potential to induce endothelial activation, ultimately leading to plaque instability (Mussbacher et al. 2022). ECs are particularly sensitive to wall shear stress (WSS), the frictional force exerted by blood flow on the endothelial lining of blood vessels. Low/oscillatory WSS is widely recognized as a catalyst for the onset/development of atherosclerosis (Mazzi et al. 2021) and is associated with lipid accumulation and plaque progression, whereas high WSS has been linked to plaque instability (Thondapu et al. 2021; Candreva et al. 2022). Areas of high WSS have been shown to colocalize with plaque ulcerations (Groen et al. 2007). While elevated WSS on its own is unlikely to cause rupture of the cap as it produces a mechanically negligible load compared to blood pressure induced tensile stress in the cap (Li et al. 2009), sustained high WSS can change plaque composition by modulating local pathobiological processes that compromise plaque integrity. For example, high WSS is known to cause endothelial dysfunction by disrupting EC alignment and promote certain cellular responses that lead to the degradation of matrix components, smooth muscle cell apoptosis and decreased matrix synthesis, resulting in reduced cap thickness (Samady et al. 2011; Loftus 2014). As a consequence, internal stress within the fibrous cap increases and tensile strength decreases, leading to a loss of mechanical stability (Finet et al. 2004). In addition to WSS magnitude, ECs have been shown to be sensitive to spatial wall shear stress gradient (WSSG) (Dolan et al. 2013). In vitro and in vivo experiments indicate that a positive WSSG, defined as increasing WSS along the flow direction, disrupts EC alignment and promotes EC dysfunction, thereby weakening the cap and increasing rupture risk (Dolan et al. 2013). Thus, positive WSSGs aggravate EC responses to high WSS (e.g., decreased EC alignment, increased apoptosis, and proliferation), while negative WSSG appears to suppress these responses (Dolan et al. 2013). High WSS and high positive WSSG are therefore regarded as important contributors to the risk of plaque destabilization.

Vulnerable plaques are metabolic active, characterized by ongoing inflammation and cellular activity. Local inflammatory processes promote the upregulation of cell adhesion molecules, such as VCAM-1 at the activated endothelial surface. The presence of increased VCAM-1 in the plaque facilitates the adhesion and recruitment of inflammatory cells such as monocytes to the site of inflammation within the plaque, which then convert to macrophages. These inflammatory cells release various enzymes and cytokines that promote the degradation of the extracellular matrix and breakdown of the fibrous cap. Thus, persistent inflammation and the enzymatic degradation of the fibrous cap can destabilize plaques and increase the likelihood of rupture. Local inflammation is therefore considered a marker for plaque vulnerability (Pasterkamp et al. 1999; Woodside et al. 2018). It has also been shown that there is a direct relationship between WSS in the plaque region and macrophage content within the plaque (Dirksen et al. 1998), as well as local VCAM-1 upregulation (Tsou et al. 2008). Therefore, estimation of VCAM-1 expression around the plaque could be used as a marker for the inflammatory status of the plaque, and in conjunction with other adverse hemodynamics-based metrics such as high WSS and high WSSG, could help identify high-risk plaques. (Weinkauf et al. 2018)

### Plaque shape as a surrogate marker for instability

Previously, we have shown that a patient’s vascular architecture strongly influences local hemodynamics including WSS (Hossain et al. 2013). Plaques of varying shapes thus can be subjected to different hemodynamic forces (Wentzel et al. 2012). Investigating the effect of lesion geometry on local hemodynamics and their relationship with eventual rupture occurrence and location can provide important insights into the role of plaque shape in plaque instability. The effect of degree of stenosis and plaque aspect ratio on local WSS has been studied in the context of predicting the location/risk of plaque formation (Wong et al. 2020; Rikhtegar et al. 2012) and understanding their role in the rupture of severely stenotic plaques (Stroud et al. 2000). The effect of longitudinal lesion asymmetry (upstream dominated and downstream dominated lesions) on WSS and axial plaque stress (APS) has been investigated using idealized single-branch coronary artery models (Choi et al. 2015) and patient-specific artery models (Lee et al. 2019) to explore rupture location and its relationship with clinical representation (Lee et al. 2017). In a recent 3D quantitative coronary angiography (3DQCA)-based study, Candreva et al. (2022) showed that WSS-based quantities such as topological shear variation index (TSVI) that accounts for WSS contraction/expansion variability could help identify lesions prone to rupture and cause MI. On the other hand, in an OCT-based computational fluid dynamics (CFD) study of a single artery segment, high WSSG was independently associated with plaque rupture (Thondapu et al. 2021). However, how local WSSG is affected by differing longitudinal lesion asymmetry within a multi-branch patient-specific coronary artery tree has not been investigated in this context. Further, few computational studies have examined the impact of plaque eccentricity on plaque instability, and none have considered plaque shapes that exhibit both eccentricity and longitudinal asymmetry. In addition, there have been no reports on the influence of 3D plaque shape on local inflammation (e.g., VCAM-1 expression), a known precursor to plaque rupture. In this work, the main objective is to develop a computational workflow to enable a comprehensive parametric study detailing the effect of 3D plaque shape features, including longitudinal asymmetry and eccentricity, on local hemodynamics and explore their implications for plaque instability. We use isogeometric finite element analysis that utilizes non-uniform rational basis splines (NURBS) to describe both the geometry *and* the solution space (Hughes et al. 2005). Isogeometric analysis has been shown to have superior accuracy and convergence in flow simulations compared to traditional finite element analysis because of its higher-order and higher-continuity basis functions (Hossain et al. 2021). Our CAD-integrated NURBS-based approach allows for a parameterized 3D plaque of arbitrary shape to be included within any branch of a patient-specific coronary artery tree model resulting in a more realistic and flexible 3D platform for such analysis. The goal is to use the computational tool developed in this work to analyze adverse hemodynamic-based metrics sensitive to plaque shape such as high WSS and high positive WSSG, along with inflammation. The findings of this study could potentially identify plaque shape features that could serve as surrogate markers of plaque instability, eventually leading to a non-invasive clinical procedure for detecting vulnerable plaques for preventive intervention.

The paper is organized as follows. In Sect. 2, we describe generation of a patient-specific coronary artery tree model, parameterization of a 3D plaque model, and the computational approach used. In Sect. 3, we present blood flow simulation results from a parametric study of different plaque shapes. Finally, in Sect. 5, we summarize and analyze the data in light of clinical observations.

## Methods

Below we describe our isogeometric analysis framework, which is particularly suitable for cardiovascular applications because of its precise and efficient geometric modeling and accurate representation of stresses and near-wall quantities (Hughes et al. 2005; Hossain et al. 2015).

### Generation of NURBS-based coronary artery model

**Fig. 2:**
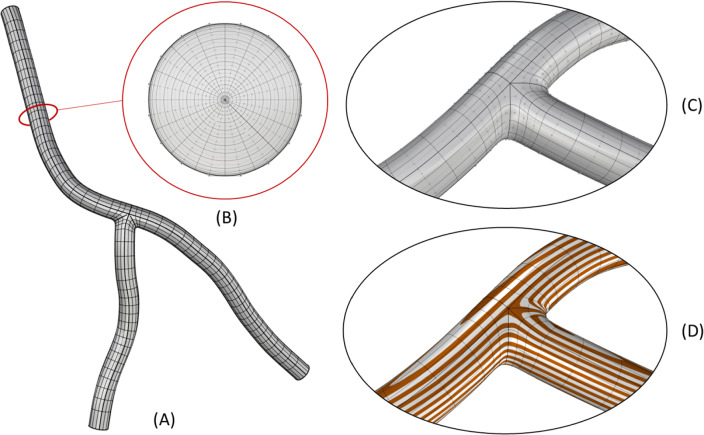
Computational mesh details. **a** The full NURBS mesh of the three-branch coronary artery model; **b** a cross section of the NURBS volume. Note, additional refinement is added towards the boundary in order to resolve the boundary layer effect in the CFD simulation; **c** a close-up of the NURBS mesh and control points near the bifurcation; **d** the continuous curvature lines to highlight the ${G}^{1}$ continuity at the patch interfaces

**Fig. 3:**
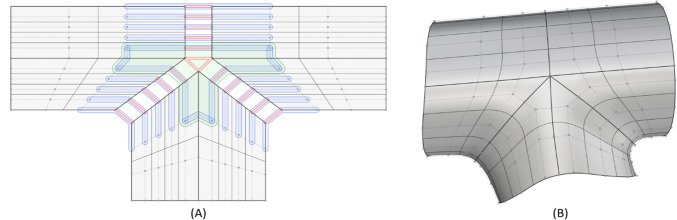
Constraints of a three-branch arterial template. The topological control point layout and constraints of a three-patch template that are necessary to join the patches with ${G}^{1}$ continuity are shown in (**a**). Red highlights indicate a coincident constraint for ${C}^{0}$ continuity. The blue and green highlights represent collinear and coplanar constraints respectively for ${G}^{1}$ continuity. The realization of the constraints illustrated in (**a**) on a portion of the three-branch model in physical space is shown in (**b**). Note, only ${G}^{0}$ continuity is achieved at the one ring spoke edges of the extraordinary point

A patient-specific geometric model of a section of the coronary artery tree is constructed from imaging data. First, the LCA (Left Coronary Artery), LCX (Left Circumflex), and LAD (Left Anterior Descending) branches are segmented from CT images. The resulting segmentation is interpolated with NURBS using a template-based approach outlined by Urick et al. (2019) (Fig. 2). The use of NURBS enables a smooth surface representation with a continuous normal vector. A 21-control point template and knot refinement near the bifurcation are used to provide additional degrees of freedom that are constrained to enforce ${G}^{1}$ continuity at patch interfaces (Fig. 3). This higher–order continuity is important for a more accurate computation of surface derivatives (e.g., WSS and WSSG), which were required for our hemodynamic analysis.

### Parameterization of 3D plaque geometry

**Fig. 4:**
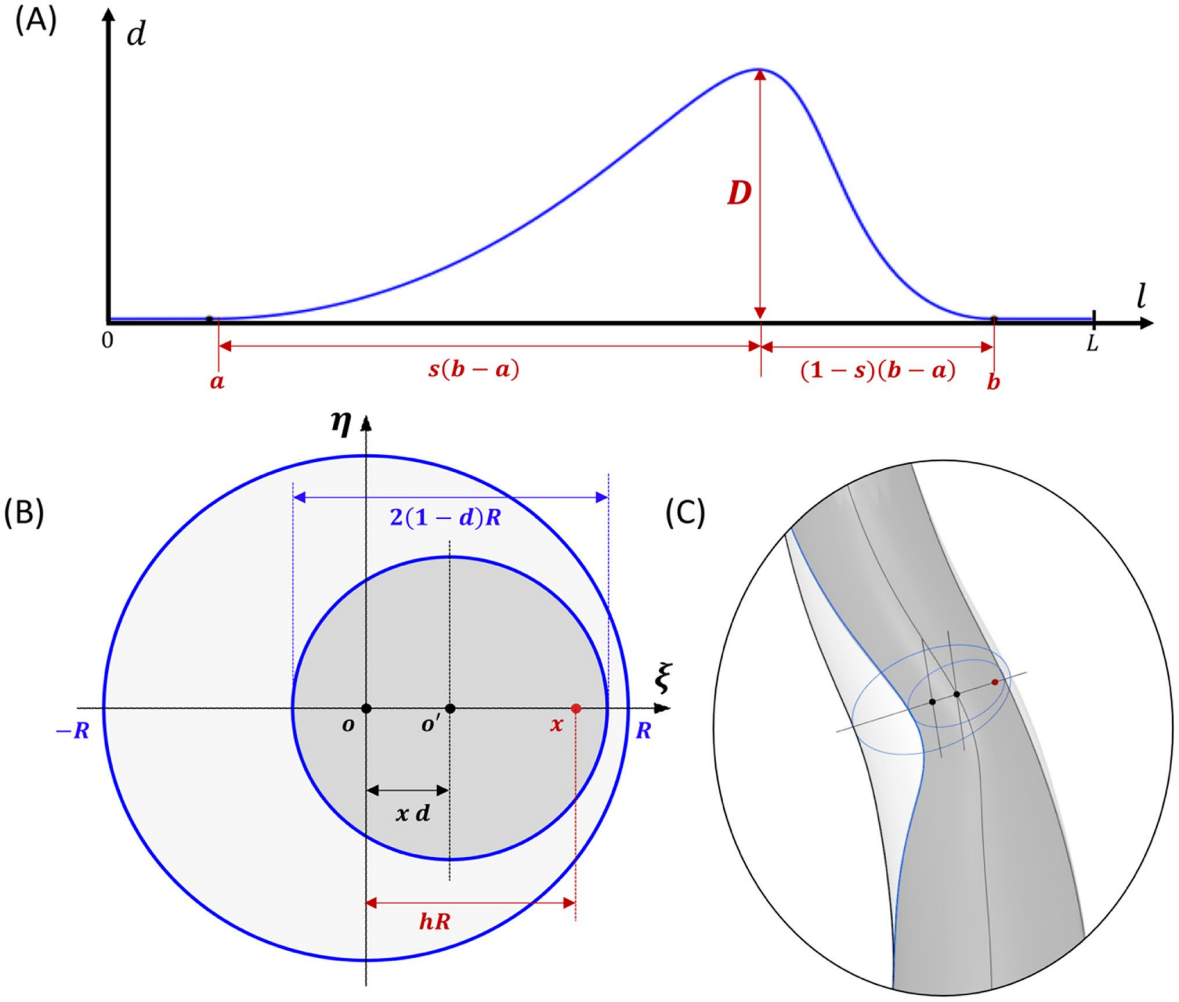
Plaque model parameterization. The plaque shape profile model that uses a cubic B-spline curve is shown in (**a**). In this model, the percent (%) stenosis, $d$, is defined as a function of length, $l$, along the centerline. $D$ is the maximum degree of stenosis; the interval $\left[a,b\right]$ controls the portion of the vessel over which the plaque has nonzero stenosis; and $s$ is the skewness parameter that shifts the position of the maximum stenosis. The parameter $h$ controls the % eccentricity for a cross-section with nominal radius $R$ in the direction of the centerline normal vector, ${\varvec{\xi}}$. A 3D example with annotations consistent with (**a**) and (**b**) is shown in (**c**)

**Fig. 5:**
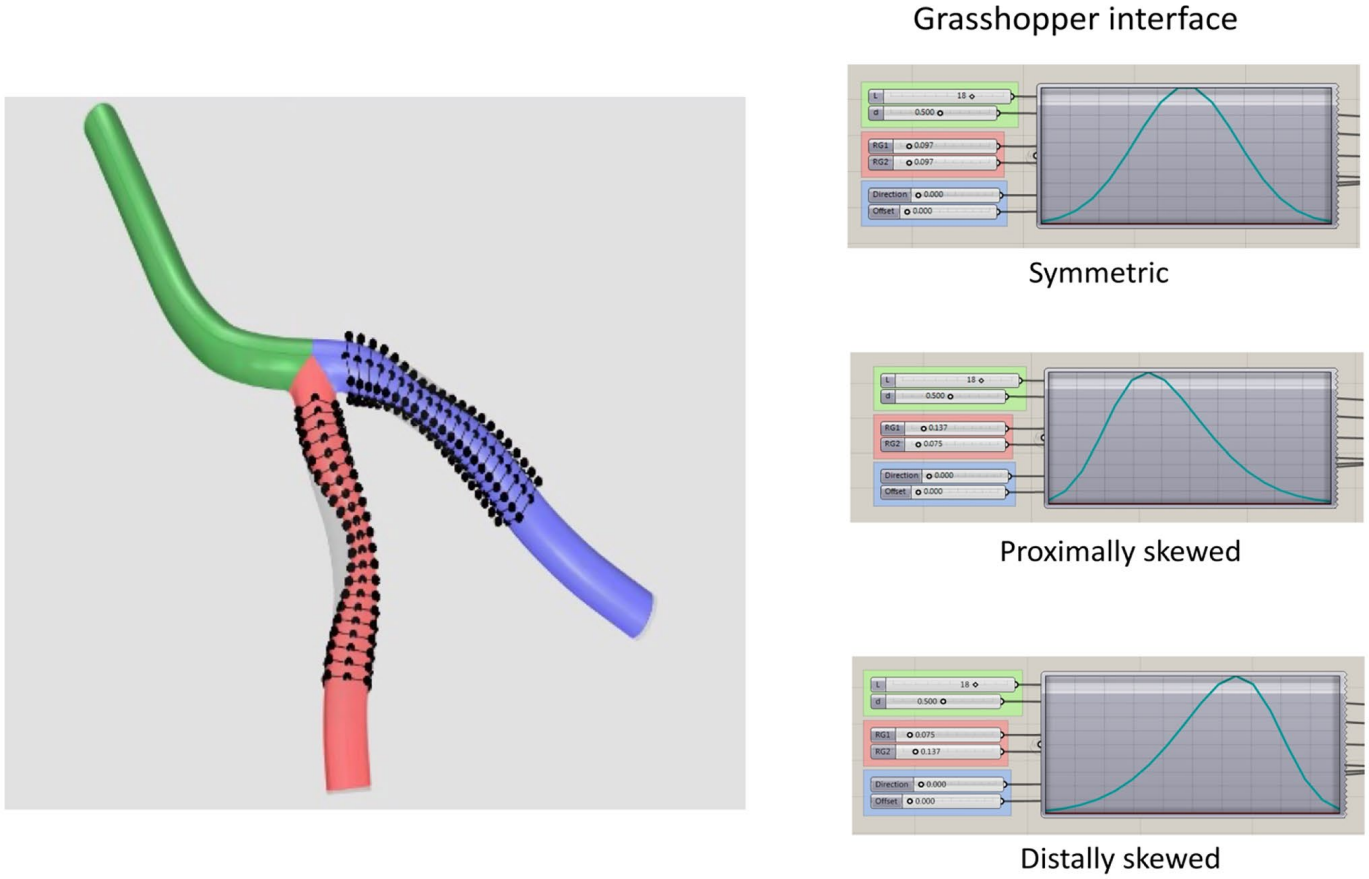
A parametric plaque model was built on top of the CAD-based patient specific modeling pipeline that enables creation of plaques of arbitrary shape within a desired branch (left panel) using a Grasshopper® interface (right panel) built within Rhinoceros®. The modeler can select a branch to build plaque into and then select a length (${L}_{{\text{p}}}$) along the selected branch. This gives the user access to the control points. A stenosis can be added by adjusting a maximum % stenosis parameter $D$. This builds a plaque along the selected length with a cubic-spline profile. The radius gradients ($R{G}_{i}$) can be adjusted by skewing the profile distally or proximally by adjusting the radius gradient parameters $R{G}_{1}$ and $R{G}_{2}$ via the skewness parameter, $s$. Finally, an eccentricity can be introduced by specifying a direction (negative or positive) and a percent offset ($h$)

A parametric plaque model is developed that modifies the vessel geometry in order to simulate the presence of atherosclerotic plaque in one or more arteries. The plaque shape is parameterized by extent, maximum stenosis, skewness, and eccentricity parameters, as illustrated in Fig. 4. The profile of the plaque shape is modeled using a cubic B-spline curve that represents the degree of stenosis, $d$, as a function of distance, $l,$ along the vessel centerline. The extent parameters, $a$ and $b$, define the length interval $\left[{a},b\right]\subset \left[0,L\right]$ over which the plaque is active, where $L$ is the length of the centerline. The stenosis parameter, $D$, controls the maximum percent (%) stenosis corresponding to the peak of the plaque profile curve. The location of the peak along the plaque profile curve is controlled by a skewness parameter,$s\in \left[\mathrm{0,1}\right]$. We say the plaque is proximally skewed, symmetric, or distally skewed when$s>0.5$,$s=0.5$, or$s<0.5$, respectively. The last parameter in the parametric plaque model controls the % eccentricity, $h\in \left[-\mathrm{1,1}\right]$. We say the plaque has negative or positive eccentricity for $h<0$ and $h>0$, respectively. When $h=0$, we say the plaque is concentric. Consider a cross section of the lumen at a position $l\in \left[a,b\right]$ along the centerline. The eccentricity is controlled by repositioning the point, ${\varvec{x}}$, about which the cross section is scaled. Let ${\varvec{o}}$ denote the centerline point, and ${\varvec{\xi}}$ the unit vector in the direction normal to the vessel centerline, as shown in Fig. 4. First, we compute ${\varvec{x}}={\varvec{o}}+h R\boldsymbol{ }{\varvec{\xi}}$ and then scale the lumen cross section by the factor $\left(1-d\left(l\right)\right)$. This process is applied to the control point frames along the extent of the plaque. The implementation of the parametric plaque model is built on top of the NURBS-based vascular modeling pipeline developed by Urick et al. (2019) using computer-aided design (CAD) software Rhino® and Grasshopper®. This allows for real-time manipulation of the vessel geometry and plaque shape by adjusting sliders that control the plaque shape parameters using the interface shown in Fig. 5. The integration of a CAD library into the model-creation algorithm provides advantages such as robustness, interoperability, accuracy, and speed.

**Fig. 6:**
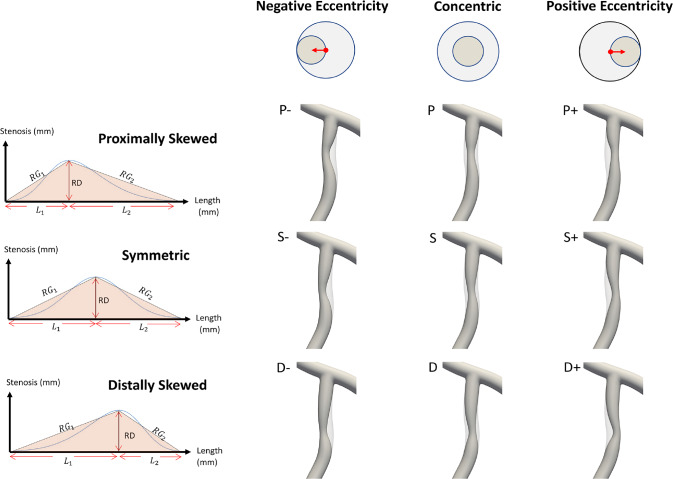
The 9 plaque shape models under consideration within the LAD branch. Left panel shows the plaque shape profile models in terms of radius gradients ($R{G}_{i}$), length of stenosis and length of plaque. The $R{G}_{i}$ is defined by the radius change over lesion length, that is the length from the lesion starting (or ending) point to minimum lumen area (MLA) location ( ${L}_{1}$ or ${L}_{2}$). Here, *RD* is the maximum stenosis in mm where $R$ is the nominal radius of the artery and *D* is % stenosis, and ${L}_{{\text{p}}}$= ${L}_{1}$+${L}_{2}$ is the total plaque length in mm. Right panel shows the 9 representative LAD plaque shape models created using the parameters reported in Table 1 for analysis

We consider two parametric plaque studies, one each for the LAD and LCX branches. For each study, we set the degree of stenosis and plaque extent to be constants while varying skewness and eccentricity. We use the parametric plaque model to instantiate 9 different plaque shapes by sampling a range of values for skewness, $s$, and eccentricity, $h$ (see Table 1 for the corresponding parameterizations). The geometries of these 9 cases for the LAD branch are shown in Fig. 6. Finally, we consider an additional study to investigate the effects of multiple plaques on hemodynamics by creating a symmetric concentric plaque in both the LAD and LCX branches.

**Table 1:** The parameterization details are reported for each of the 9 cases considered in the parametric studies

Case	Plaque Model Description	${L}_{p}$ (mm)	$D$(%)	$h$	RG_1_	RG_2_
S	Symmetric, Concentric	18	0.5	0	.097	.097
P	Proximally Skewed, Concentric	18	0.5	0	.137	.075
D	Distally Skewed, Concentric	18	0.5	0	.075	.137
S-	Symmetric, Neg. Eccentricity	18	0.5	− 1	.097	.097
S +	Symmetric, Pos. Eccentricity	18	0.5	+ 1	.097	.097
P-	Proximally Skewed, Neg. Eccentricity	18	0.5	− 1	.137	.075
P +	Proximally Skewed, Pos. Eccentricity	18	0.5	+ 1	.137	.075
D-	Distally Skewed, Neg. Eccentricity	18	0.5	− 1	.075	.137
D +	Distally Skewed, Pos. Eccentricity	18	0.5	+ 1	.075	.137

The plaque length ${L}_{p}$ and max stenosis, $D$ are constant across all cases. The skewness for each case is reported in terms of the radius gradients (RGs), where ${{\text{RG}}}_{1}=\frac{{\text{RD}}}{{\text{sLp}}}$, and ${{\text{RG}}}_{2}=\frac{{\text{RD}}}{\left(1-{\text{s}}\right){\text{Lp}}}$, and plaque eccentricity in terms of $h$. The plaque length ${L}_{p}$ and RG values are chosen according to data presented in (Lee et al. 2017)

### Governing equations and solution strategy

**Fig. 7:**
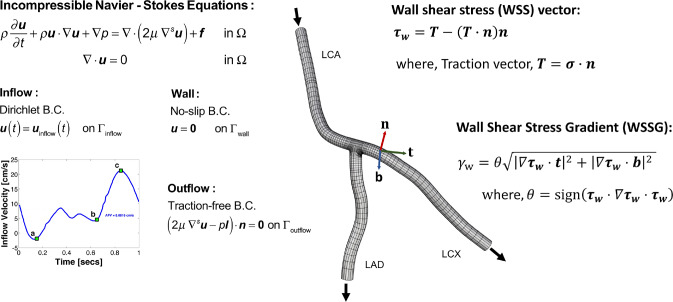
The problem setup. The hexahedral NURBS computational mesh of a three-branch coronary artery tree model (nominal case) used in simulation is shown. Here, the total number of elements is 36,864 and total number of unique control points is 50,463. The governing equations subjected to boundary conditions (B.C.s) are also presented, where **u** represents velocity, *p* is the pressure, $f$ is the body force (set to zero), $\mu$ is dynamic viscosity, $\rho$ is density, $t$ is time, and ${\varvec{n}}$ is the unit normal. A pulsatile inflow condition is prescribed at left coronary artery (LCA) inlet. A no-slip B.C. is imposed on the vessel wall, and a traction-free B.C. is set at the outlets of the left anterior descending (LAD) and left circumflex (LCX) branches. Wall shear stress (WSS) vector, ${{\varvec{\tau}}}_{{\varvec{w}}}$, and wall shear stress gradient (WSSG), ${\gamma }_{w}$, are computed from the traction vector ***T***, where ${\varvec{\sigma}}$ is the stress tensor. Here, ${\varvec{t}}$ is the unit vector in the direction of ${{\varvec{\tau}}}_{{\varvec{w}}}$ and ${\varvec{b}}$ is perpendicular to ${\varvec{t}}$ and ${\varvec{n}}$

Blood flow within a patient-specific vascular network is simulated by adopting a continuum-based approach. Figure 7 provides an overview of the problem setup. The details of the governing equations and the numerical procedures are reported elsewhere (Hossain et al. 2013; Hossain 2009; Horn et al. 2022). Briefly, blood is modeled as an incompressible Newtonian fluid with a density (*ρ*) of 1060kg/m^3^ and a dynamic viscosity (*μ*) of 0.003 N-s/m^2^. Blood flow is assumed to be governed by the unsteady Navier–Stokes equations for incompressible flow. A time-dependent inflow velocity waveform (Johnston et al. 2006) is prescribed at the inlet with a parabolic profile assuming a fully developed flow (Hossain et al. 2015), a no-slip boundary condition is set at the rigid wall, and a traction-free outflow boundary condition is specified at the branch outlets. For numerical approximation of the solution, finite element based isogeometric analysis is used with quadratic NURBS to describe the geometry *and* the solution space (Hughes et al. 2005). A residual-based variational multiscale method (Bazilevs et al. 2007) is applied to solve the system of equations, utilizing a Newton–Raphson procedure with multi-stage predictor–corrector algorithm applied at each time step. The generalized−$\alpha$ method (Jansen et al. 2000) is used for time stepping.

### WSS and WSSG parameters

Let ${\varvec{S}}\left({u}^{0},{u}^{1}\right)$ be a ${C}^{1}$ continuous surface. Then, the covariant basis vectors $\left\{{{\varvec{g}}}_{0}, {{\varvec{g}}}_{1}\right\}$ are given by the partial derivatives of the surface mapping with respect to its parameters:

$${{\varvec{g}}}_{\alpha }=\frac{\partial {\varvec{S}}}{\partial {u}^{\alpha }}$$

the contravariant basis can be computed by the following:

$${{\varvec{g}}}^{\alpha }={g}^{\alpha \beta }{{\varvec{g}}}_{\beta }$$

$$\left[{g}^{\alpha \beta }\right]={\left[{g}_{\alpha \beta }\right]}^{-1}$$

$${g}_{\alpha \beta }={{\varvec{g}}}_{\alpha }\cdot {{\varvec{g}}}_{\beta }$$

where Greek indices $\alpha , \beta$ take on the values 0 and 1, and repeated indices are summed. Let ${\varvec{T}}\left({u}^{0},{u}^{1}\right)$ be a continuous traction vector field on ${\varvec{S}}$ computed from a post processing of a CFD analysis. The traction vector field is then projected on the quadratic ${C}^{1}$ basis. Then, the WSS vector, ${{\varvec{\tau}}}_{{\varvec{w}}}$*,* is defined as the component of ${\varvec{T}}$ in the tangent space of ${\varvec{S}}$.

$${{\varvec{\tau}}}_{{\varvec{w}}}=\left({{\varvec{g}}}^{\alpha }\cdot {\varvec{T}}\right){{\varvec{g}}}_{\alpha }$$

The WSSG tensor, $\nabla {{\varvec{\tau}}}_{{\varvec{w}}}$, is computed by projecting the spatial gradient of wall shear stress vector onto the tangent space of ${\varvec{S}}$.

$$\nabla {{\varvec{\tau}}}_{{\varvec{w}}}=\left({{\varvec{g}}}^{\alpha }\cdot \frac{\partial {{\varvec{\tau}}}_{{\varvec{w}}}}{\partial {u}^{\beta }}\right){{\varvec{g}}}_{\alpha }\otimes {{\varvec{g}}}^{\beta }$$

Note, in this work, the curvature term that arises from applying the derivative operator on the normal component of the traction vector is neglected (Cherubini et al. 2015). In the literature, hemodynamic WSSG is defined by a signed scalar measure of $\nabla {{\varvec{\tau}}}_{{\varvec{w}}}$ that is computed according to a Pythagorean norm of directional derivatives of ${{\varvec{\tau}}}_{{\varvec{w}}}$ in two orthogonal directions (Lei et al. 1996). Let ${\varvec{t}}$ denote the unit vector in the direction of ${{\varvec{\tau}}}_{{\varvec{w}}}$. Let $\mathbf{b}$ denote the unit vector orthogonal to both $\mathbf{t}$ and the unit surface normal, ${\varvec{n}}$. Then, the WSSG, ${\gamma }_{w}$, can be computed by evaluating the following.

$${\gamma }_{w}=\theta \sqrt{{\Vert \nabla {{\varvec{\tau}}}_{{\varvec{w}}}\cdot {\varvec{t}}\Vert }^{2}+{\Vert \nabla {{\varvec{\tau}}}_{{\varvec{w}}}\cdot {\varvec{b}}\Vert }^{2}}$$

where $$\theta $$ is:

$$\theta ={\text{sign}}\left({{\varvec{\tau}}}_{{\varvec{w}}}\cdot \nabla {{\varvec{\tau}}}_{{\varvec{w}}}\cdot {{\varvec{\tau}}}_{{\varvec{w}}}\right)$$

For each simulation, the CFD solution is post processed to compute the traction field, ${\varvec{T}}={\varvec{\sigma}}\cdot {\varvec{n}}$ at the artery wall, which is then used to compute ${{\varvec{\tau}}}_{{\varvec{w}}}$ and ${\gamma }_{w}$. We refer to ${{\varvec{\tau}}}_{{\varvec{w}}}$ as the WSS vector and $\tau_{w} : = \left\| {{\varvec{\tau}}_{{\varvec{w}}} } \right\|$ as the WSS magnitude.

### Estimation of vascular VCAM-1 expression

**Fig. 8:**
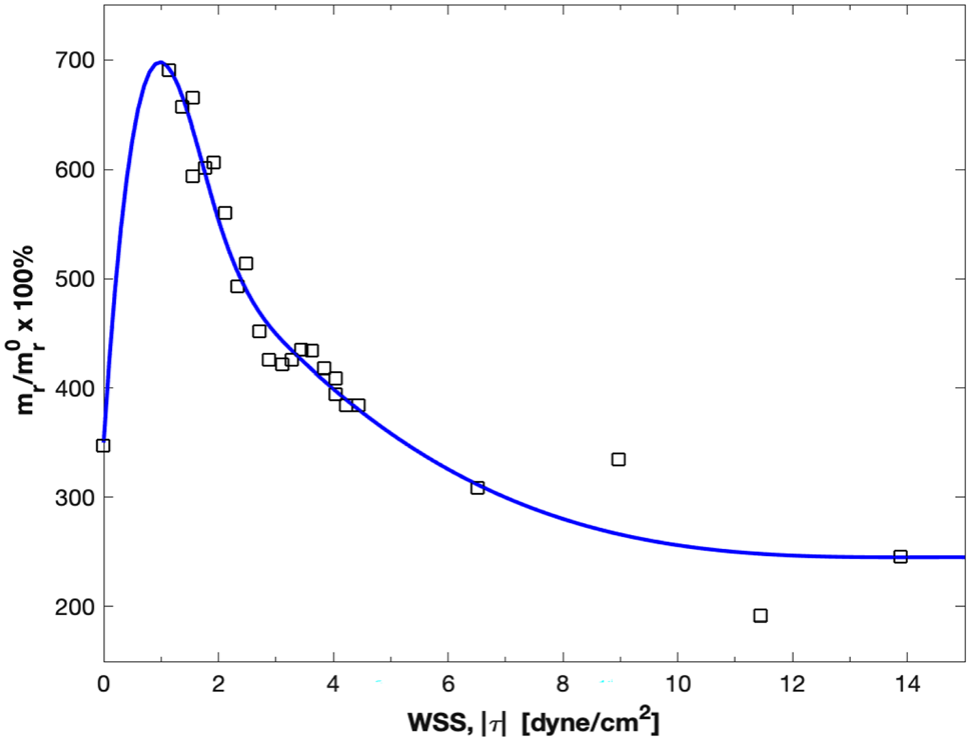
Estimation of inflammatory response to local wall shear stress. Vascular cell adhesion molecule (VCAM-1) surface density vs. wall shear stress relationship determined by curve fitting to in-vitro data for Tumor Necrotic Factor (TNF)-α stimulated expression. Here squares denote experimental data for VCAM-1 and the solid line represents the corresponding fitted data. The quantities are reported as percent (%) of unstimulated VCAM-1 expression under static conditions (m_r_/m_r_^0^). Addition of TNF-α under static conditions stimulated up-regulation of VCAM-1 by 350%

Through an in vitro study, Tsou and co-workers (Tsou et al. 2008) correlated overexpression of inflammatory vascular molecules such as VCAM-1 to local WSS. By fitting a cubic spline to that experimental data, we developed a phenomenological model for estimating VCAM-1 surface density as a function of local WSS (Hossain et al. 2014). The in vitro data and the corresponding fitted curves are presented in Fig. 8. We use this phenomenological model to quantify the vascular distribution of VCAM-1 to assess the inflammatory status of plaques.

## Results

**Fig. 9:**
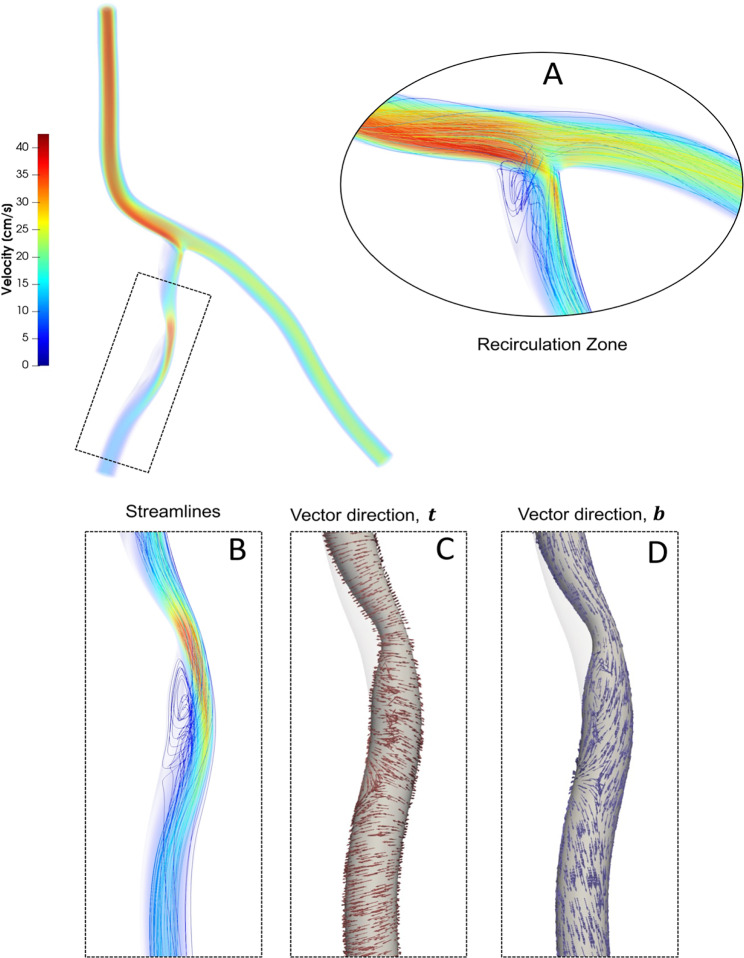
Blood flow simulation results for a representative LAD plaque case, proximally skewed with positive eccentricity (P+), in terms of streamlines colored with velocity magnitude. Insets show prominent recirculation zones (**a**, **b**); WSS vector direction ${\varvec{t}}$ (**c**); and vector direction ${\varvec{b}}$ (**d**)

Blood flow simulations are performed for the patient-specific coronary artery tree model generated from imaging data. Nine different plaque shape models are considered for the LAD branch and the LCX branch ( a total of 18 cases) of the coronary artery tree and compared with the nominal (no-plaque) case. An additional case is considered where two symmetric concentric plaques are concurrently located in the downstream branches of the coronary artery tree: one in LAD and one in LCX (“SS”). Figure 9 shows the blood flow simulation results for a representative distally skewed LAD plaque with a positive eccentricity (“D + ”). Flow recirculation zones are observed near the bifurcation, at the intersection of the LCA and LAD branches, as well as downstream of the throat (maximum stenosis) that is the minimum lumen area (MLA). While a 50/50 flow split is seen at the bifurcation for the nominal (“N”) case, with the presence of a stenosis, more flow is directed to the non-stenosed artery at peak systole, as expected (Vignon-Clementel et al. 2010), resulting in a 65/35 flow split.

### Effect of plaque shape on WSS and WSSG

**Fig. 10:**
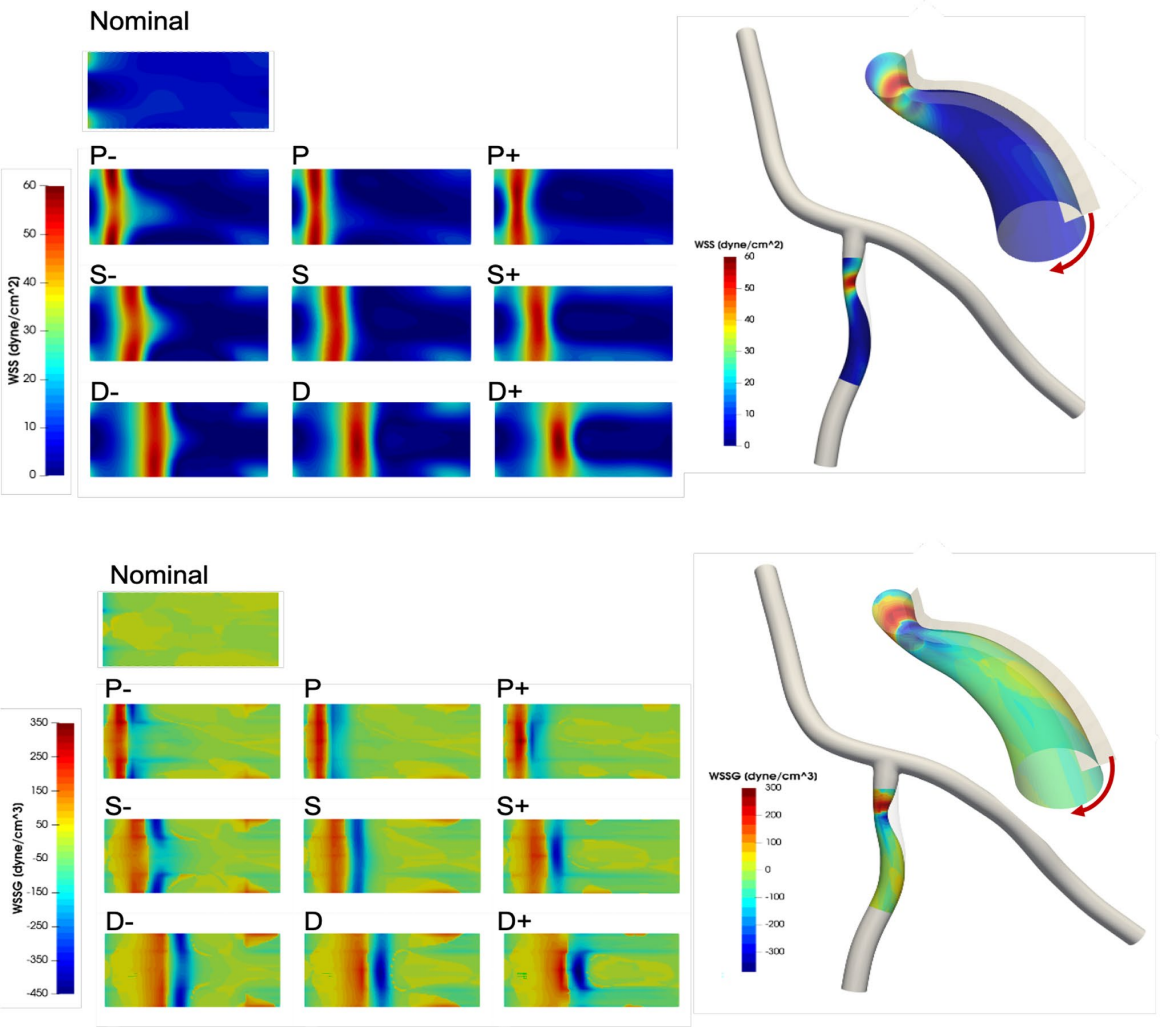
WSS magnitude (top panel) and WSSG value (bottom panel) distribution for the 9 different LAD plaque models under consideration. Here, “P” denotes a proximally skewed, “S” denotes a symmetric, and “D” denotes a distally skewed plaque. The negative and positive signs indicate the direction of eccentricity. Here, nominal represents the no-plaque case

**Fig. 11:**
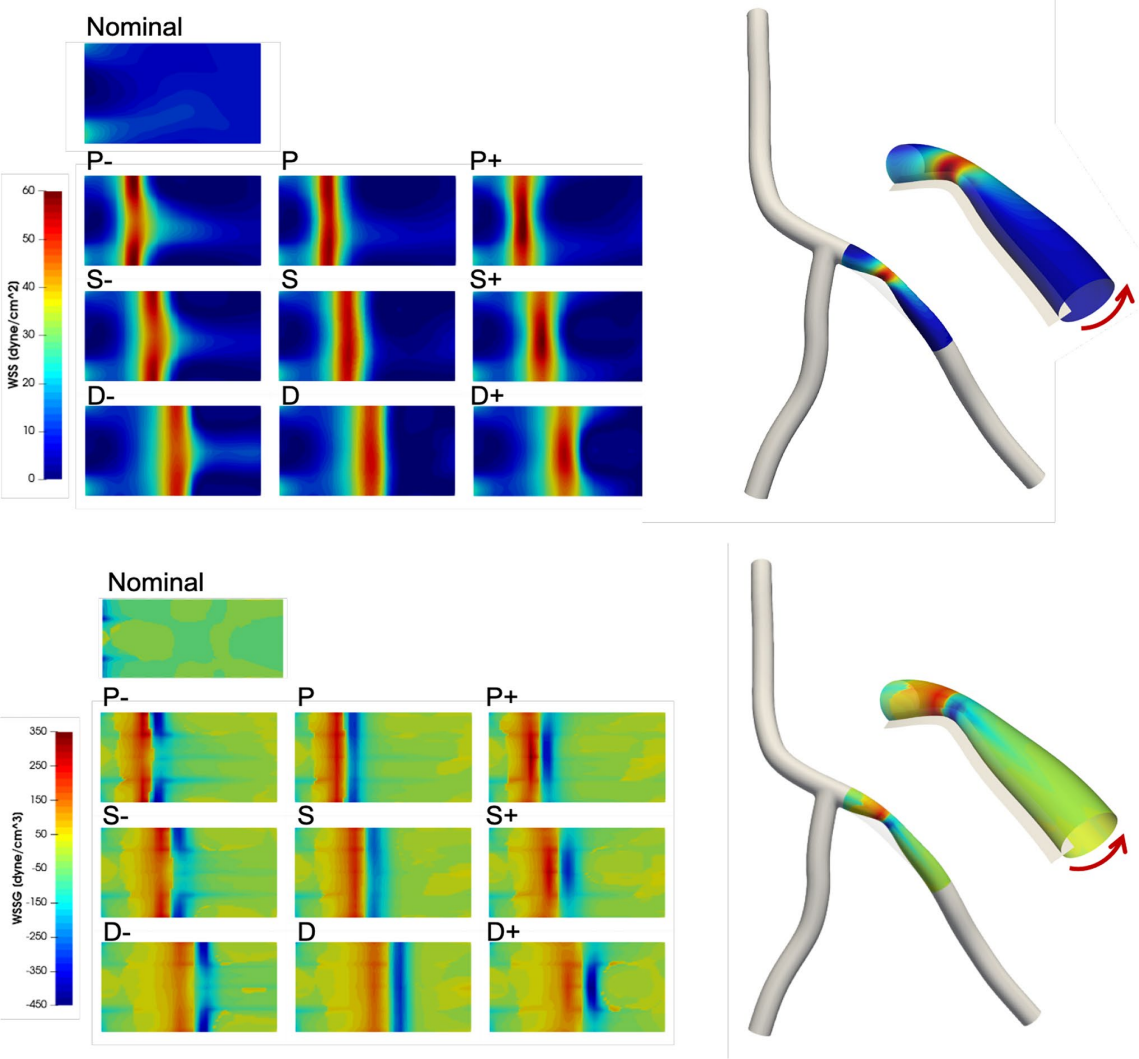
WSS magnitude (top panel) and WSSG (bottom panel) distribution for the 9 different LCX plaque shape models under consideration. Here, “P” denotes a proximally skewed, “S” denotes a symmetric, and “D” denotes a distally skewed plaque. The negative and positive signs indicate the direction of eccentricity. Here, nominal represents the no-plaque case

**Fig. 12:**
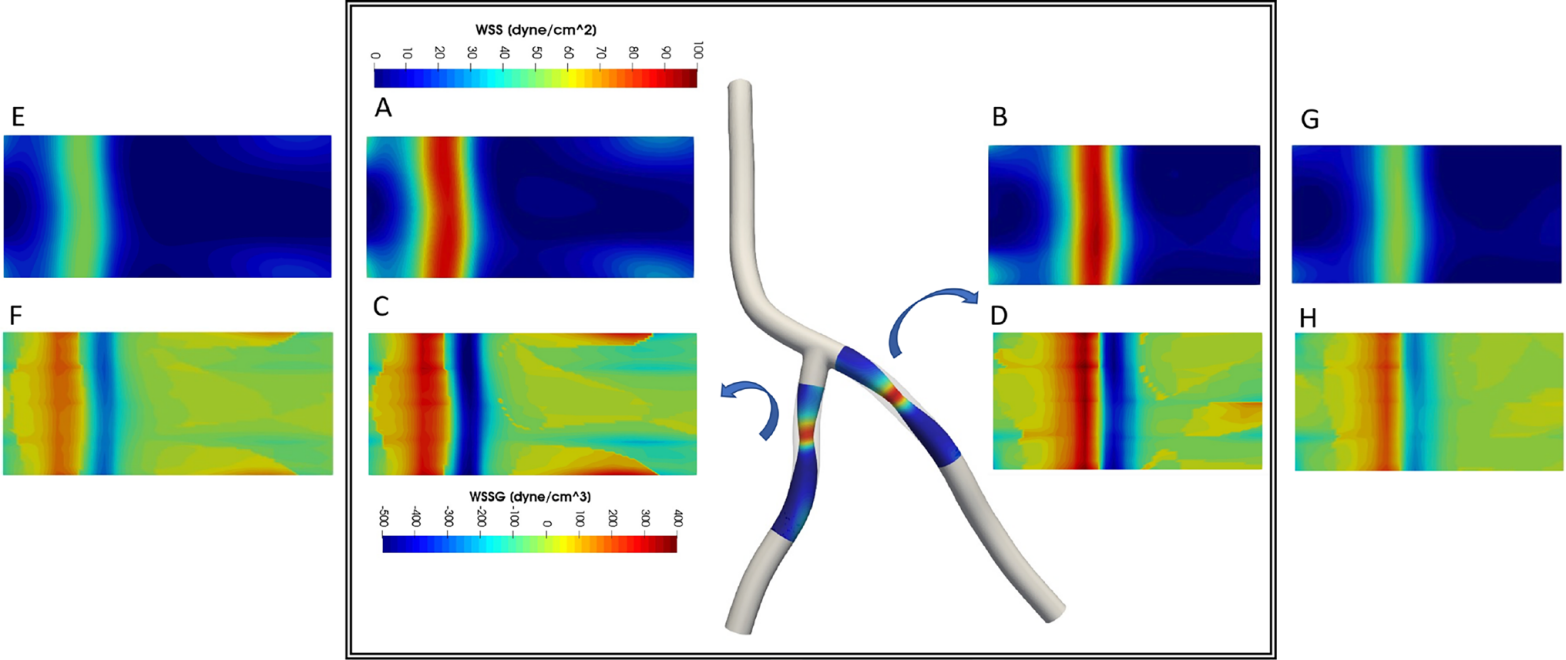
Multiple plaques versus a single plaque. The inner panel shows WSS (top) and WSSG (bottom) distribution patterns within the LAD (a and c) and LCX (b and d) branches when there is a symmetric plaque in both LAD and LCX branches ( “SS”). These results are compared with corresponding single plaque (“S”) cases in the LAD (e and f) and LCX (g and h)

**Fig. 13:**
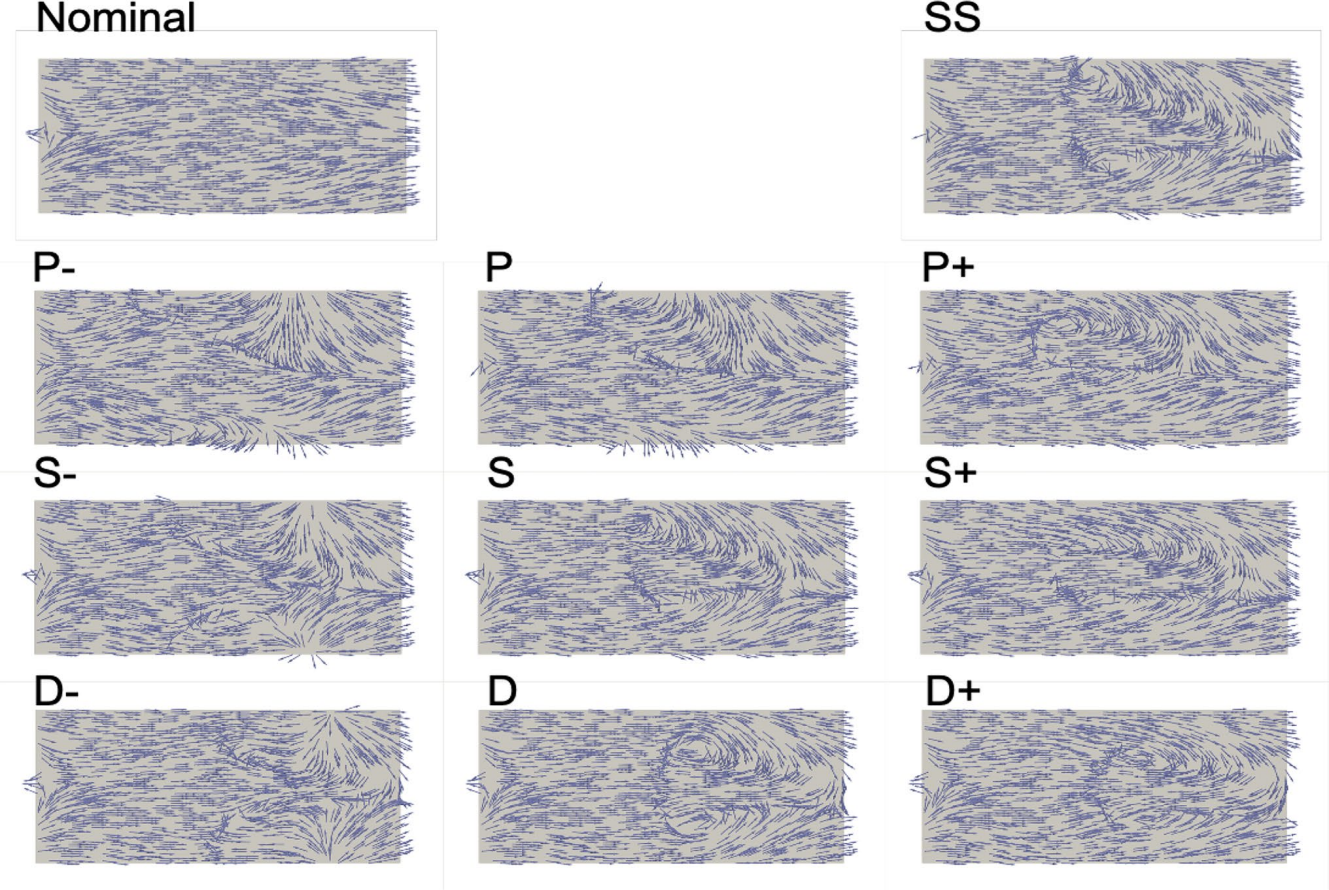
WSS vector directions (not scaled by magnitude) at peak systole for the 9 different LAD plaque shape models under consideration. Here, “P” denotes a proximally skewed, “S” denotes a symmetric, and “D” denotes a distally skewed plaque. The negative and positive signs indicate the direction of eccentricity. Nominal (“N”) represents the no-plaque case and “SS” represents the multi-plaque case when there is a symmetric plaque in both LAD and LCX branches (only the LAD plaque is shown here)

Near wall quantities of interest, WSS and WSSG are computed for each plaque configuration. Figures 10 and 11 show WSS magnitude and WSSG distribution around the plaque at peak systole for the 9 LAD and LCX plaque shapes considered, respectively. The artery segment containing the plaque is unrolled to show the 2D surface. The presence of plaque increases max WSS seen within the LAD and the LCX by a factor of 1.72 and 2.12 on average, respectively, when compared with the nominal (“N”) case. A band of higher WSS generally appears close to the MLA for all the plaque shapes studied. The WSS distribution patterns for plaques with either positive eccentricity (“P + ”, “S + ”, “D + ”) or negative eccentricity (“P-”, “S-”, “D-”) are different from those for the concentric plaques (“P”, “S”, and “D”). For the plaques with a negative eccentricity, the band of higher WSS extends further downstream of the MLA giving it a more convex shape compared to the concentric cases. For plaques with a positive eccentricity that band assumes more of a concave shape downstream of MLA. Figure 12 presents the WSS and WSSG distribution patterns at peak systole for the multi-plaque case (“SS”) considered and compared with those for the corresponding single symmetric centered (“S”) LAD and LCX plaque cases analyzed earlier. While the distribution patterns for the LAD and LCX plaques remain similar, the presence of two plaques instead of one markedly increases the maximum WSS magnitude from 53 to 85 dyne/cm^2^ and 59 to 92 dyne/cm^2^, respectively. WSSG results exhibit a similar behavior and the maximum WSSG value in the LAD and LCX increased from 212 to 320 dyne/cm^3^ and 236 to 391 dyne/cm^3^, respectively. Table 2 reports the maximum WSS magnitude and maximum WSSG values for all the cases analyzed. Figure 13 depicts WSS vector directions around the LAD plaque for the 9 plaque shapes considered, along with the multi-plaque case (“SS”) and the nominal (“N”) case. Flow recirculation zones are seen downstream of all plaques, and their location depends on the presence and extent/direction of plaque eccentricity and longitudinal asymmetry (skewness). The presence of a plaque in the neighboring LCX branch does not appear to affect flow directions in the LAD branch in the multi-plaque case (“SS”).

**Table 2:** Maximum WSS magnitude ($\Vert {{\varvec{\tau}}}_{w}\Vert$) in dyne/cm^2^ and maximum positive scalar WSSG gradient (${\gamma }_{w}$) in dyne/cm^3^ seen in the plaque models considered

Case	Plaque Model Description	LAD		LCX	
		Max WSS	Max WSSG	Max WSS	Max WSSG
N	Nominal	31	47	25	78
S-	Symmetric, Neg. Eccentricity	54	207	59	295
S	Symmetric, Concentric	53	197	55	236
S +	Symmetric, Pos. Eccentricity	54	218	56	287
P-	Proximally Skewed, Neg. Eccentricity	57	334	61	342
P	Proximally Skewed, Concentric	57	268	57	279
P +	Proximally Skewed, Pos. Eccentricity	60	291	60	325
D-	Distally Skewed, Neg. Eccentricity	58	298	55	200
D	Distally Skewed, Concentric	56	238	52	173
D +	Distally Skewed, Pos. Eccentricity	55	212	51	189
SS	Symmetric-Symmetric	85	320	91	391

**Fig. 14:**
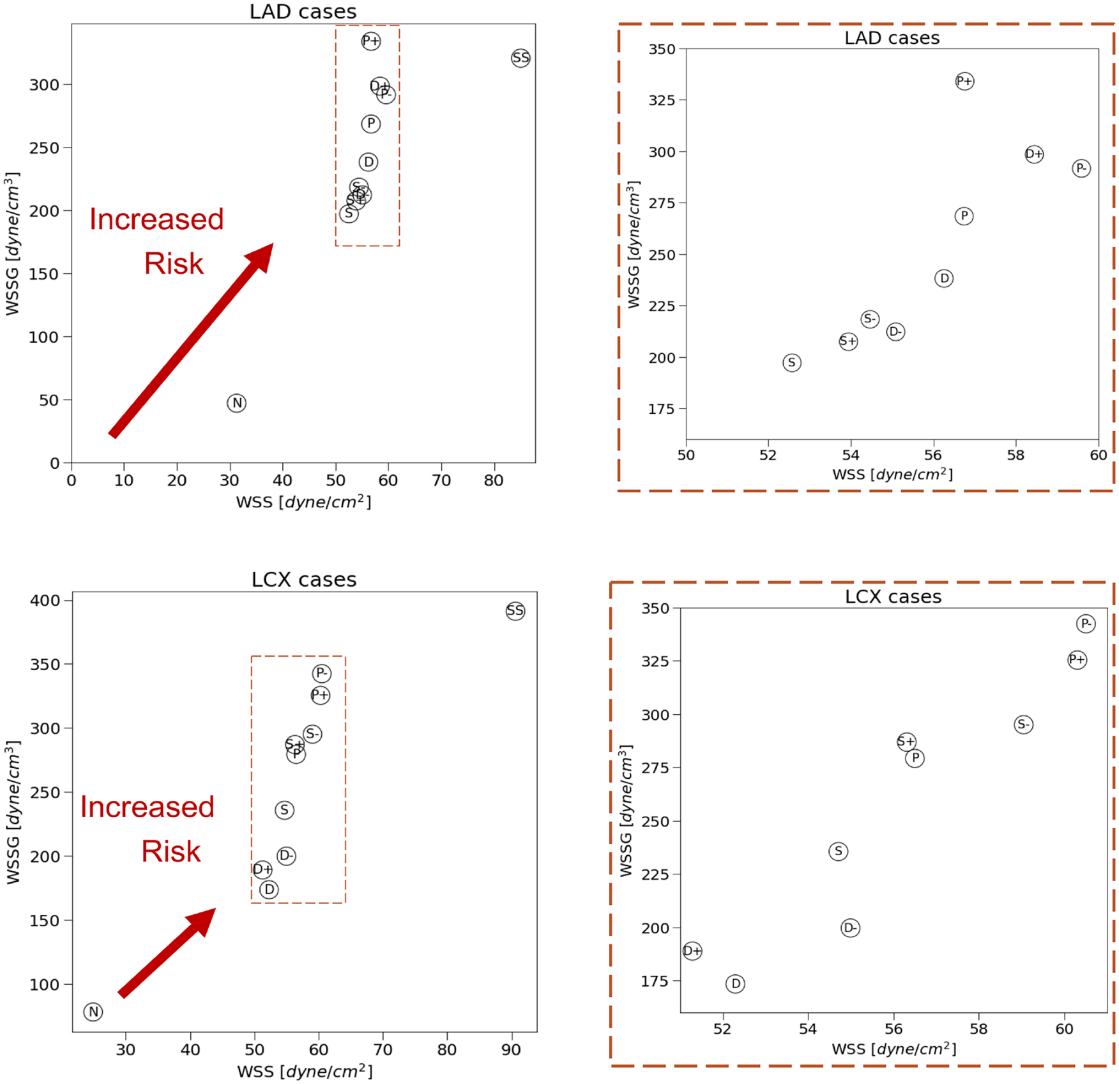
A graphical summary of the LAD plaque (top row) and LCX plaque (bottom row) results reported in Table 2. The scatter plots on the left show the maximum WSS magnitude and WSSG values observed in all 9 plaque shape models along with the multi-plaque (“SS”) case and the no-plaque nominal (“N”) case. The objective is to identify the plaque shape cases that have the most vulnerable combination of high WSS and high positive WSSG that are deemed at a higher risk of rupture that others. The scatter plots on the right show zoomed in views of the highlighted region on the left

A graphical summary of these results is shown in Fig. 14. The scatter plots take into consideration the maximum WSS magnitude and maximum positive WSSG value for all plaque cases studied to help identify the specific plaque shape(s) with the most vulnerable combination of high WSS and high positive WSSG signifying a higher risk of rupture. Among the LAD plaque shapes analyzed, proximally skewed eccentric plaques (“P + ” and “P- “) appear to be more prone to rupture, while symmetric plaques (“S + ”, “S”, “S- “) are more stable. Among the LCX plaque shapes considered, once again, proximally skewed eccentric plaques are at a higher risk of rupture, while distally skewed (“D + ”, “D”, “D- “) plaques are less vulnerable than others.

### Effect of plaque shape on VCAM-1 expression

**Fig. 15:**
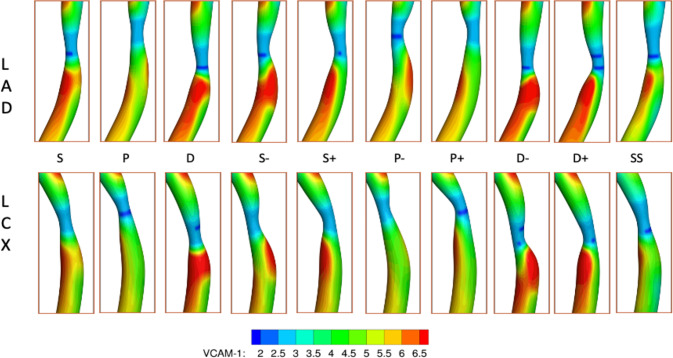
Spatial distribution of inflammatory marker VCAM-1 (*m*_*r*_/*m*_*r*_^0^) for the 9 plaque shape cases considered for the LAD (top row) and LCX (bottom row) branches

From the blood flow simulation results, mean (time averaged) WSS is computed throughout the coronary artery tree. Then, utilizing the phenomenological model correlating VCAM-1 expression to WSS, the surface density of VCAM-1 (*m*_*r*_) is estimated with respect to unstimulated expression under static conditions (*m*_*r*_^0^), representing the healthy state. Figure 15 shows the distribution of VCAM-1 surface expressions (*m*_*r*_ /*m*_*r*_^0^) in the plaque region as a marker for the inflammatory status within the plaque for all the cases considered. Spatially heterogeneous distribution of local WSS triggers distinctly different inflammatory responses for VCAM-1.

## Summary and discussion

There is an overwhelming need for developing a noninvasive clinical procedure for identifying unstable vulnerable plaques before they rupture and cause heart attacks. Local hemodynamics are known to influence plaque instability. Existing literature, bolstered by a recent study (Thondapu et al. 2021), indicates that while low WSS is linked to lipid accumulation, high WSS is associated with the location of acute rupture. High WSS is believed to play a dual role in this context, acting directly as a biomechanical stressor exerted "against" the plaque and, indirectly, by altering its composition and rendering the plaque more susceptible to rupture (e.g., weakening the fibrous cap) (Russo et al. 2023). High positive WSSG exacerbates these adverse effects of high WSS and is independently associated with rupture (Thondapu et al. 2021). Therefore, high WSS and high positive WSSG are considered key risk factors for acute plaque destabilization.

A preliminary study on a simplified cylindrical coronary artery tube model indicates that plaque shape strongly influences local WSS and WSSG. Results show that while the maximum velocity remains largely unaffected by plaque shape for the same degree of stenosis, maximum WSS and maximum WSSG vary by as much as 14.9% and 45.4%, respectively, among the four shapes considered (results not shown here). Based on these findings, we hypothesized that plaque shape could potentially serve as a surrogate marker for plaque instability. To facilitate testing of this hypothesis, we developed a computational toolset to enable a comprehensive parametric study detailing the effect of 3D plaque shape on local hemodynamics in a patient-specific coronary artery tree. The objective is to identify plaque shape(s) with the most vulnerable combination of high WSS and high positive WSSG.

Our CAD-based vascular modeling tool allows us to efficiently create plaques of arbitrary shape and size within a coronary artery tree. We make use of a NURBS-based isogeometric analysis framework (Hughes et al. 2005), which enables differentiable geometric representations with greater accuracy and smoothness compared to the traditional finite-element analysis. In this work, we use multi-patch NURBS with higher-order geometric continuity at patch interfaces. Moreover, the parametric definition of NURBS meshes enables automatic boundary layer refinement near the arterial wall, which is crucial for numerical stability and a more accurate computation of near-wall hemodynamic quantities like WSS. In order to investigate the effects of plaque shape, we introduce a parametric 3D plaque modeler that makes modifications to the original patient-specific geometry to simulate the presence of an atherosclerotic plaque. The parametric plaque modeler operates directly on the patient-specific multi-patch NURBS geometries by modifying control point positions. This approach enables smooth geometric modifications to be made that maintain higher-order surface continuity for the hemodynamic analysis. Alternative approaches for modifying patient-specific vascular geometries include morphMan® and sVMorph® (Pham et al. 2022; Bergersen et al. 2020). However, these programs are not equipped to operate on parametric surfaces such as NURBS.

In a previous work, Lee et al. (2017) studied a population of 125 patients with known plaque rupture history and examined the relationships among longitudinal lesion geometry (proximally skewed vs. distally skewed), axial plaque stress (APS) acting on the plaque, location of plaque rupture, and clinical presentation. The mean plaque length and radius gradients ($RG{\text{s}}$) reported in that study are used in this work to create plaques of different shapes for our analysis, as outlined in the Methods section. The $RG$ is defined by the radius change over lesion length (Fig. 6). In addition to the longitudinal asymmetry studied by Lee et al. (2017), we consider the effect of plaque eccentricity (about the artery centerline) and constructed seven representative plaque shape models for each LAD and LCX branch. A multi-plaque case is also taken into consideration. The effect of plaque shape and location on local WSS and WSSG is analyzed and compared with corresponding results for the nominal (“N”) case.

Our blood flow analysis of the coronary artery tree indicates that local hemodynamic metrics WSS and WSSG are not only sensitive to longitudinal asymmetry or skewness, but also plaque eccentricity. The results suggest that plaque eccentricity increases rupture risk for all plaque shapes with the exception of the distally skewed LAD plaque with negative eccentricity (“D- “). Interestingly, according to an IVUS study of 106 patients, ruptured coronary plaques that ultimately causes an acute coronary syndrome are typically eccentric (Yamagishi et al. 2000). Among the LAD and LCX single plaque cases considered, proximally skewed eccentric plaques have the most vulnerable combination of high positive WSSG and high WSS. This is consistent with clinical observations made by Lee et al. (2017) who reported that proximally skewed lesions are most common (69.5%), and led to more acute events. Note, our preliminary study with the idealized single artery cylindrical model suggested that a symmetric eccentric plaque has more adverse hemodynamic characteristics and therefore has a higher risk of rupture than a proximally skewed eccentric plaque, which is not the case for the patient-specific model. This emphasizes the importance of incorporating a more realistic patient-specific multi-branch coronary artery tree, as opposed to a simplified single coronary artery in such analyses. Blood flow simulation of the coronary artery tree with a symmetric-concentric plaque in both LAD and LCX (“SS”) revealed a 1.6-fold higher peak WSS magnitude and WSSG values compared to their single plaque counterparts. This aligns with the results reported in the study conducted by Glaser et al. (2005), which correlated the presence of multiple lesions in an MI population with higher incidence of cardiac events.

In our analysis, maximum WSS magnitude occurs near the MLA, and maximum WSSG value generally occurs further upstream of the max WSS location, regardless of LAD plaque shape and location. This suggests a higher risk of an upstream or even an MLA rupture. These findings are intriguing because in a recent study of human carotid atherosclerotic plaques, ruptures predominantly occurred in the proximal and most stenotic (MLA) regions but not in the distal region (Sun et al. 2023). Lee et al. (2017) also previously reported that 98.5% of proximally skewed lesions, which are significantly more prevalent than distally skewed lesions, had an upstream rupture. On the other hand, distally skewed plaques, although far less common, consistently had a downstream rupture in that study. Our analysis of maximum WSS and maximum WSSG did not predict a higher risk of downstream rupture for the distally skewed plaques, or any plaques for that matter. Additional metrics might be needed to predict a rarely occurring downstream rupture (Sun et al. 2023).

We then explored the inflammatory status of each plaque shape considered by estimating overexpression of inflammatory cell adhesion molecule VCAM-1. For all cases, VCAM-1 upregulation is minimal in the MLA region. A noticeably higher VCAM-1 upregulation is observed just downstream of the MLA. Maximum VCAM-1 surface density in the downstream area is on average 1.6-fold higher compared to that in the nominal case and can be up to 4.5-fold higher relative to the MLA region, indicating a higher risk of downstream rupture. This effect appears to be less pronounced upstream of the MLA. The location and extent of this VCAM-1 rich region downstream of the MLA vary with the presence and direction of plaque eccentricity, and the maximum generally occurs around the plaque shoulder region, the transition zone between the fibrous cap and the underlying plaque core that is often characterized by the accumulation of inflammatory cells. Interestingly, plaque rupture is also frequently colocalized with inflammation of the cap and shoulder region (Pasterkamp et al. 1999). Our analysis thus suggests that in conjunction with patient-specific data, a combination of adverse hemodynamic factors including high WSS and high positive WSSG along with inflammation (VCAM-1) may need to be considered for predicting the occurrence and location of plaque rupture.

There are a few limitations of this study. Patient-specific in vivo flow rate measurements were unavailable for the coronary artery tree imaged for model creation. A representative pulsatile inflow velocity waveform (Johnston et al. 2006) is used for inflow boundary condition, and for simplicity, a traction-free outflow boundary condition is implemented. The traction-free boundary condition neglects the downstream hemodynamic resistance of the truncated vessels. As a result, flow split at the bifurcation can be influenced by the lengths of outlet branches (McElroy and Keshmiri 2018) and the presence of a severe stenosis (Vignon-Clementel et al. 2010). While no outflow boundary condition has been proven superior to others, reduced order models such as the Windkessel model are considered more realistic (Shimano et al. 2019). For example, it has been shown that a 50/50 mean (time-averaged) flow split at the bifurcation can be preserved with an impedance boundary condition with more flow directed to the non-stenosed artery at peak systole, followed by decreased flow at diastole (Vignon-Clementel et al. 2010). However, these reduced order models have their disadvantages including difficulty obtaining flow and pressure data at required locations, uncertainty of model parameters, more complex numerical implementation, and higher computational costs. Although it has been suggested that the power law strategy (e.g., Murray’s law) achieves a good balance between cost and physiological reality (Shimano et al. 2019), given that the traction-free boundary condition is unlikely to significantly deviate from Murray's law (Shimano et al. 2019), we deemed it to be a reasonable initial approximation for the purposes of this study. Further, considering the simulation results for the different plaque shape cases, with the same degree (nonsignificant) of stenosis and plaque length, were contrasted with the nominal case, we believe the relative impact of different plaque shape geometry on local WSS and WSSG was captured reasonably well, which was a major goal of this study. A more physiologically realistic outflow boundary condition will be incorporated in future work. Another limitation worth noting is that the results presented in this work may not apply to a broader patient population because a single patient-specific coronary artery geometry was considered.

In the future, we plan to perform a retrospective study involving a large population with known plaque rupture history and location. We will create patient-specific models from noninvasive imaging data (e.g., coronary CTA) and characterize lesion geometry according to a low-dimensional representation such as the parameterization used in this paper (radius gradient, eccentricity, etc.), or one that is learned using artificial intelligence techniques. Using statistical methods, we will then identify (i) correlations between shape-based metrics and adverse hemodynamic metrics such as high WSS, high WSSG, inflammation and (ii) shape-based predictors of plaque instability that can be used to improve risk stratification and treatment for patients. Additional hemodynamic metrics such as time–averaged and time variance quantities will also be considered.

In summary, we developed a CAD-based parametric plaque modeling framework that provides a flexible platform for analyzing the effect of 3D plaques of arbitrary shape, in conjunction with patient-specific attributes, on hemodynamics-based factors that impact plaque stability. Results of our parametric study suggest that proximally skewed eccentric plaque shape has a higher risk of rupture, and that plaque shape could be indicative of plaque instability. The image-based computational modeling tool developed in this work may enable identification of plaque shape-based metrics as surrogate markers for plaque vulnerability. If confirmed in a planned future study involving a large patient population, this could ultimately lead to a clinical procedure for noninvasive assessment of plaque rupture risk.
